# What Drives Academic Data Sharing?

**DOI:** 10.1371/journal.pone.0118053

**Published:** 2015-02-25

**Authors:** Benedikt Fecher, Sascha Friesike, Marcel Hebing

**Affiliations:** 1 Internet-enabled Innovation, Alexander von Humboldt Institute for Internet and Society, Berlin, Germany; 2 Research Infrastructure, German Institute for Economic Research, Berlin, Germany; 3 German Socio-Economic Panel, German Institute for Economic Research, Berlin, Germany; University of York, UNITED KINGDOM

## Abstract

Despite widespread support from policy makers, funding agencies, and scientific journals, academic researchers rarely make their research data available to others. At the same time, data sharing in research is attributed a vast potential for scientific progress. It allows the reproducibility of study results and the reuse of *old* data for *new* research questions. Based on a systematic review of 98 scholarly papers and an empirical survey among 603 secondary data users, we develop a conceptual framework that explains the process of data sharing from the primary researcher’s point of view. We show that this process can be divided into six descriptive categories: *Data donor, research organization, research community, norms, data infrastructure*, and *data recipients*. Drawing from our findings, we discuss theoretical implications regarding knowledge creation and dissemination as well as research policy measures to foster academic collaboration. We conclude that research data cannot be regarded as knowledge commons, but research policies that better incentivise data sharing are needed to improve the quality of research results and foster scientific progress.

## Introduction

The accessibility of research data has a vast potential for scientific progress. It facilitates the replication of research results and allows the application of *old* data in *new* contexts [1,2]. It is hardly surprising that the idea of shared research data finds widespread support among academic stakeholders. The European Commission, for example, proclaims that access to research data will boost Europe’s innovation capacity. To tap into this potential, data produced with EU funding should to be accessible from 2014 onwards [3]. Simultaneously, national research associations band together to promote data sharing in academia. The *Knowledge Exchange Group*, a joint effort of five major European funding agencies, is a good example for the cross-border effort to foster a culture of sharing and collaboration in academia [4]. Journals such as *Atmospheric Chemistry and Physics, F1000Research, Nature*, or *PLoS One*, increasingly adopt data sharing policies with the objective of promoting public access to data.

In a study among 1,329 scientists, 46% reported they do not make their data electronically available to others [5]. In the same study, around 60% of the respondents, across all disciplines, agreed that the lack of access to data generated by others is a major impediment to progress in science. Though the majority of the respondents stem from North America (75%), the results point to a striking dilemma in academic research, namely the mismatch between the general interest and the individual’s behavior. At the same time, they raise the question of what exactly prevents researchers from sharing their data with others.

Still, little research devotes itself to the issue of data sharing in a comprehensive manner. In this article we offer a cross-disciplinary analysis of prevailing barriers and enablers, and propose a conceptual framework for data sharing in academia. The results are based on a) a systematic review of 98 scholarly papers on the topic and b) a survey among 603 secondary data users who are analyzing data from the German Socio-Economic Panel Study (hereafter SOEP). With this paper we aim to contribute to research practice through policy implications and to theory by comparing our results to current organizational concepts of knowledge creation, such as commons-based peer production [6] and crowd science [7]. We show that data sharing in today’s academic world cannot be regarded a knowledge commons.

The remainder of this article is structured as follows: first, we explain how we methodologically arrived at our framework. Second, we will describe its categories and address the predominant factors for sharing research data. Drawing from these results, we will in the end discuss theory and policy implications.

## Methodology

**Fig 1:**
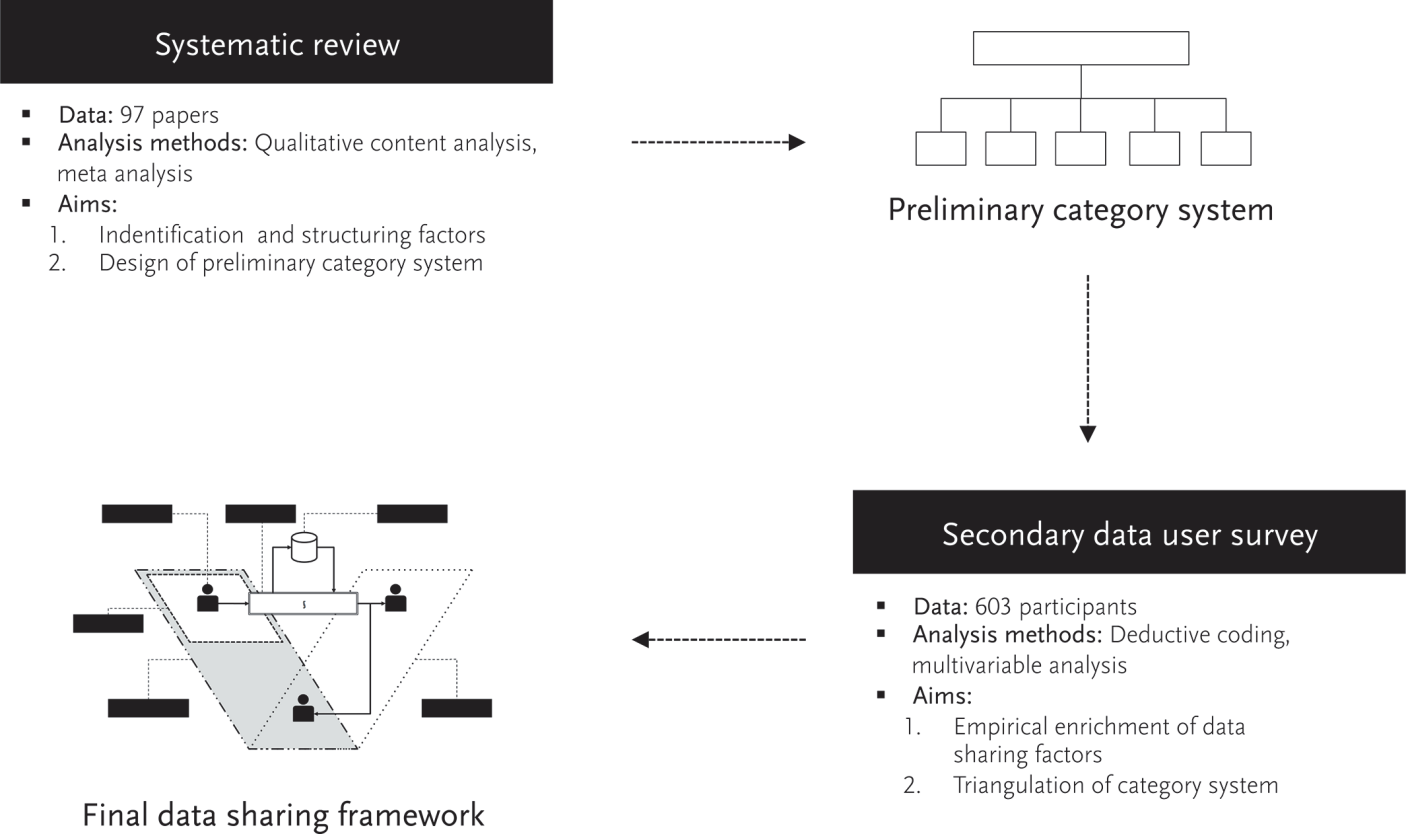
Research Methodology.

In order to arrive at a framework for data sharing in academia, we used a systematic review of scholarly articles and an empirical survey among secondary data users (*SOEP User survey*). The first served to design a preliminary category system, the second to empirically revise it. In this section, we delineate our methodological approach as well as its limitations. Fig. 1 illustrates the research methodology.

### 2.1 Systematic Review

Systematic reviews have proven their value especially in evidence based medicine [8]. Here, they are used to systematically retrieve research papers from literature databases and analyze them according to a pre-defined research question. Today, systematic reviews are applied across all disciplines, reaching from educational policy [9,10] to innovation research [11]. In our view, a systematic review constitutes an elegant way to start an empirical investigation. It helps to gain an exhaustive overview of a research field, its leading scholars, and prevailing discourses and can be used to produce an analytical spadework for further inquiries.

### 2.1.1 Retrieval of Relevant Papers

**Fig 2:**
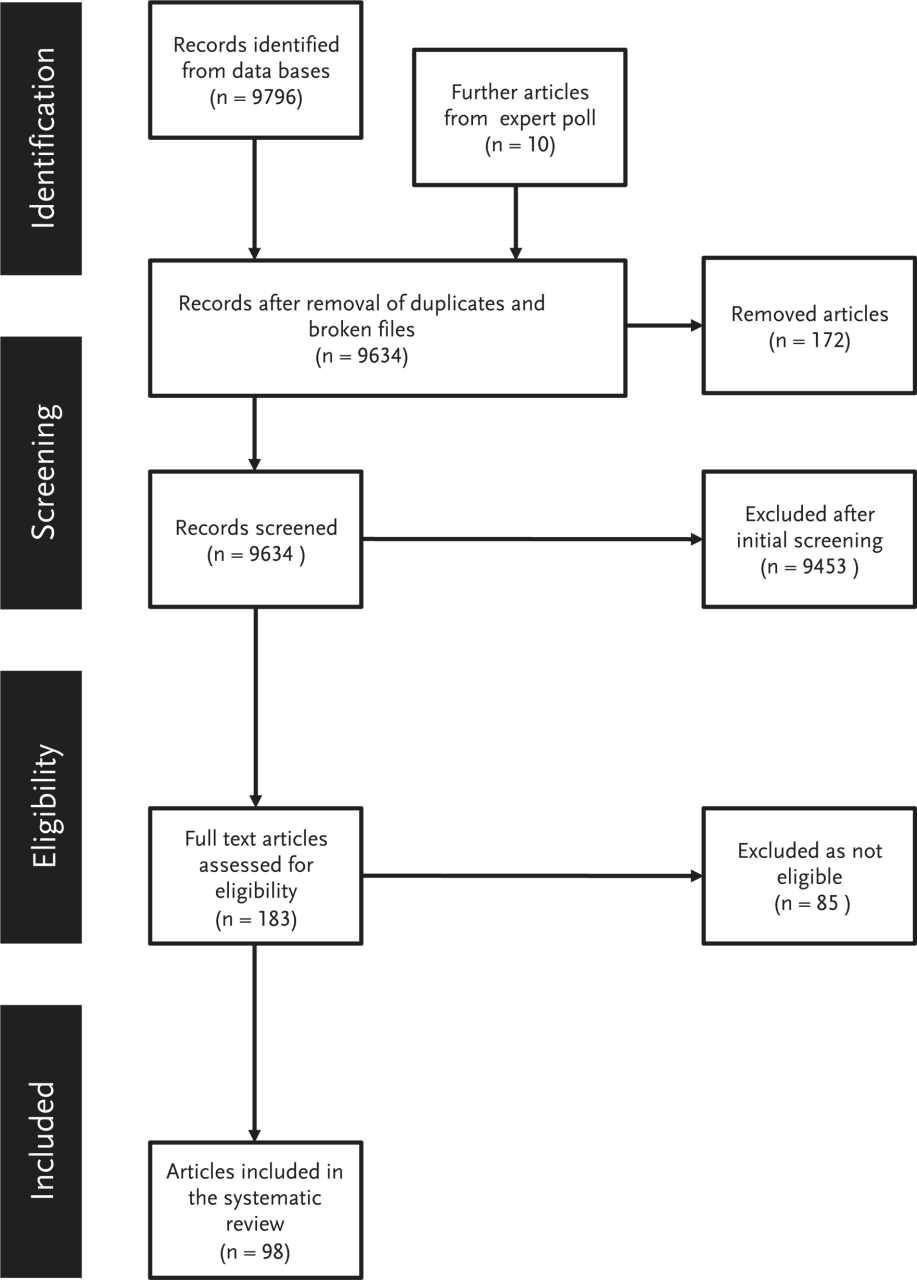
Flowchart selection process.

In order to find the relevant papers for our research intent, we defined a research question (*Which factors influence data sharing in academia*?) as well as explicit selection criteria for the inclusion of papers [12]. According to the criteria, the papers needed to address the perspective of the primary researcher, focus on academia and stem from defined evaluation period. To ensure an as exhaustive first sample of papers as possible, we used a broad basis of multidisciplinary data banks (see Table 1) and a search term (“*data sharing”)* that generated a high number of search results. We did not limit our sample to research papers but also included for example discussion papers. In the first sample we included every paper that has the search term in either title or abstract. Fig. 2 summarizes the selection process of the papers.

**Table 1:** Paper and databases of the final sample.

**Database**	**Papers in analysis sample**
Ebsco	Butler, 2007; Chokshi et al., 2006; De Wolf et al., 2005 (also JSTOR, ProQuest); De Wolf et al., 2006 (also ProQuest); Feldman et al., 2012; Harding et al., 2011; Jiang et al., 2013; Nelson, 2009; Perrino et al., 2013; Pitt and Tang, 2013; Teeters et al., 2008 (also Springer)
JSTOR	Anderson and Schonfeld, 2009; Axelsson and Schroeder, 2009 (also ProQuest); Cahill and Passamano, 2007; Cohn, 2012; Cooper, 2007; Costello, 2009; Fulk et al., 2004; Constable and Guralnick, 2010; Linkert et al., 2010; Ludman et al., 2010; Myneni and Patel, 2010; Parr, 2007; Resnik, 2010; Rodgers and Nolte, 2006; Sheather, 2009; Whitlock et al., 2010; Zimmerman, 2008
PLOS	Alsheikh-Ali et al,. 2011; Chandramohan et al., 2008; Constable et al., 2010; Drew et al., 2013; Haendel et al., 2012; Huang et al., 2013; Masum et al., 2013; Milia et al., 2012; Molloy, 2011; Noor et al., 2006; Piwowar, 2011; Piwowar et al., 2007; Piwowar et al., 2008; Savage and Vickers, 2009; Tenopir et al., 2011; Wallis et al., 2013; Wicherts et al., 2006;
ProQuest	Acord and Harley, 2012; Belmonte et al., 2007; Edwards et al., 2011; Eisenberg, 2006; Elman et al., 2010; Kim and Stanton, 2012; Nicholson and Bennett, 2011; Rai and Eisenberg, 2006; Reidpath and Allotey, 2001(also Wiley); Tucker, 2009
ScienceDirect	Anagnostou et al., 2013; Brakewood and Poldrack, 2013; Enke et al., 2012; Fisher and Fortman, 2010; Karami et al., 2013; Mennes et al., 2013; Milia et al.; 2013; Parr and Cummings, 2005; Piwowar and Chapman, 2010; Rohlfing and Poline, 2012; Sayogo and Pardo, 2013; Van Horn and Gazzaniga, 2013; Wicherts and Bakker, 2012
Springer	Albert, 2012; Bezuidenhout, 2013; Breeze et al., 2012; Fernandez et al., 2012; Freymann et al., 2012; Gardner et al., 2003; Jarnevich et al., 2007; Jones et al., 2012; Pearce and Smith, 2011; Sansone and Rocca-Serra, 2012
Wiley	Borgman, 2012; Dalgleish et al., 2012; Delson et al., 2007; Eschenfelder and Johnson, 2011; Haddow et al., 2011; Hayman et al., 2012; Huang et al., 2012; Kowalczyk and Shankar, 2011; Levenson, 2010; NIH, 2002; NIH, 2003; Ostell, 2009; Piwowar, 2010; Rushby, 2013; Samson, 2008; Weber, 2013
From the expert poll	Campbell et al., 2002; Cragin et al., 2010; Overbey, 1999; Sieber, 1988; Stanley and Stanley, 1988

The evaluation period spanned from December 1^st^ 2001 to November 15^th^ 2013, leading to a pre-sample of 9796 papers. We read the abstracts of every paper and selected only those that a) address data sharing in academia and b) deal with the perspective of the primary researcher. In terms of intersubjective comprehensibility, we decided separately for every paper if it meets the defined criteria [13]. Only those papers were included in the final analysis sample that were approved by all three coders (yes/yes/yes in the coding sheet). Papers that received a no from every coder were dismissed; the others were discussed and jointly decided upon. The most common reasons for dismissing a paper were thematic mismatch (e.g., paper focusses on commercial data), and quality issues (e.g., a letter to the editor). Additionally, we conducted a small-scale expert poll on the social network for scientists *ResearchGate*. The poll resulted in five additional papers, three of which were not published in the defined evaluation period. We did, however, include them in the analysis sample due to their thematic relevance. In the end, we arrived at a sample of 98 papers. Table 1 shows the selected papers and the database in which we found them.

### 2.1.2 Sample description

**Fig 3:**
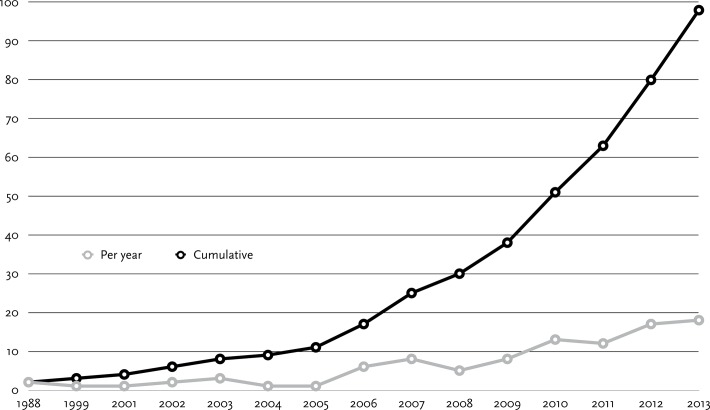
Papers in the sample by year.

The 98 papers that made our final sample come from the following disciplines: Science, technology, engineering, and mathematics (60 papers), humanities (9), social sciences (6), law (1), interdisciplinary or no disciplinary focus (22). The distribution of the papers indicates that data sharing is an issue of relevance across all research areas, above all the STEM disciplines. The graph of our analysis sample (see Fig. 3) indicates that academic data sharing is a topic that has received a considerable increase in attention during the last decade.

Further, we analyzed the references that the 98 papers cited. Table 2 lists the most cited papers in our sample and provides an insight into which articles and authors dominate the discussion. Two of the top three most cited papers come from the journal PLoS One. Among the most cited texts, [14] is the only reference that is older than 2001.

**Table 2:** Most cited within the sample.

**Reference**	**# Citations**
Piwowar, H.A., Day, R.S., Fridsma, D.B. 2007. Sharing detailed research data is associated with increased citation rate. PLoS ONE, 2(3): e308.	15
Campbell, E.G., Clarridge, B.R., Gokhale, M., Birenbaum, L., Hilgartner, S., Holtzman, N.A., Blumenthal, D. 2002. Data withholding in academic genetics: evidence from a national survey. JAMA, 287(4): 473–480.	15
Savage, C.J., Vickers, A.J. 2009. Empirical study of data sharing by authors publishing in PloS journals. PLoS ONE 4(9): e7078.	12
Feinberg, S.E., Martin, M.E., Straf, M.L. 1985. Sharing Research Data. Washington, DC: National Academy Press.	9
NIH National Institutes of Health. 2003. Final NIH statement on sharing research data Available.	9
Wicherts, J.M., Borsboom, D., Kats, J., Molenaar, D. 2006. The poor availability of psychological research data for reanalysis. American Psychologist, 61(7): 726–728.	9
Nelson, B. 2009. Data sharing: empty archives. Nature, 461: 160–163.	8
Tenopir, C., Allard, S., Douglass, K., Aydinoglu, A.U., Wu, L., Read, E., Manoff, M., Frame, M. 2011. Data Sharing by Scientists: Practices and Perceptions. PloS ONE 6(6): e21101.	8
Gardner, D., Toga, A.W., Ascoli, G.A., Beatty, J.T., Brinkley, J.F., Dale, A.M., Fox, P.T., Gardner, E.P., George, J.S., Goddard, N., Harris, K.M., Herskovits, E.H., Hines, M.L., Jacobs, G.A., Jacobs, R.E., Jones, E.G, Kennedy, D.N., Kimberg, D.Y., Mazziotta, J.C., Perry L. Miller, Mori, S., Mountain, D.C., Reiss, A.L., Rosen, G.D., Rottenberg, D.A., Shepherd, G.M., Smalheiser, N.R., Smith, K.P., Strachan, T., Van Essen, D.C., Williams, R.W., Wong, S.T.C. 2003. Towards effective and rewarding data sharing. Neuroinformatics, 1(3): 289–295.	8
Borgman C.L. 2007. Scholarship in the Digital Age: Information, Infrastructure, and the Internet. Cambridge, MA: MIT Press.	8
Whitlock M.C. 2011. Data Archiving in Ecology and Evolution: Best Practices. Trends in Ecology and Evolution, 26(2): 61–65.	7

### 2.1.3 Preliminary Category System

In a consecutive, we applied a qualitative content analysis in order to build a category system and to condense the content of the literature in the sample [15,16]. We defined the analytical unit compliant to our research question and copied all relevant passages in a *CSV* file. After, we uploaded the file to the data analysis software *NVivo* and coded the units of analysis inductively. We decided for inductive coding as it allows building categories and establishing novel interpretative connections based on the data material, rather than having a conceptual pre-understanding. The preliminary category system allows allocating the identified factors to the involved individuals, bodies, regulatory systems, and technical components.

### 2.2 Survey Among Secondary Data Users

To empirically revise our preliminary category system, we further conducted a survey among 603 secondary data users that analyze data from the German Socio-Economic Panel (SOEP). We specifically addressed secondary data users because this researcher group is familiar with the re-use of data and likely to offer informed responses.

The SOEP is a representative longitudinal study of private households in Germany [17]. It is conducted by the German Institute for Economic Research. The data is available to researchers through a research data centre. Currently, the SOEP has approximately 500 active user groups with more than 1,000 researchers per year analyzing the data. Researchers are allowed to use the data for their scientific projects and publish the results, but must neither re-publish the data nor syntax files as part of their publications.

The SOEP User Survey is a web-based usability survey among researchers who use the panel data. Beside an annually repeated module of socio-demographic and service related questions, the 2013 questionnaire included three additional questions on data sharing (see Table 3). The annual questionnaire includes the Big Five personality scale according to Richter et al. [18] that we correlated with the willingness to share (see *3.1. Data Donor*). When working with panel surveys like the SOEP, researchers expend serious effort to process and prepare the data for analysis (e.g., generating new variables). Therefore the questions were designed more broadly, including the willingness to share analysis scripts.

It has to be added that different responses could have resulted if the willingness to share data sets and the willingness to share analysis scripts/code would be seperated in the survey. The web survey was conducted in November and December 2013, resulting in 603 valid response cases—of which 137 answered the open questions *Q2* and *Q3*. We analyzed the replies to these two open questions by applying deductive coding and using the categories from the preliminary category system. We furthermore used the replies to revise categories in our category system and add empirical evidence.

**Table 3:** Questions for secondary data users.

**Q1** We are considering giving SOEP users the possibility to make their baskets and perhaps also the scripts of their analyses or even their own datasets available to other users within the framework of SOEPinfo. Would you be willing to make content available here?
*Yes, I would be willing to make my own data and scripts publicly available*
*Yes, but only on a controlled-access site with login and password*
*Yes, but only on request*
*No*
**Q2** What would motivate you to make your own scripts or data available to the research community? (open answer)
**Q3** What concerns would prevent you from making your own scripts or data available to the research community? (open answer)

The respondents are on average 37 years old, 61% of them are male. Looking at the distribution of disciplines among the researchers in our sample, the majority works in economics (46%) and sociology/social sciences (39%). For a German study it is not surprising that most respondents are German (76%). Nevertheless, 24% of the respondents are international data users. The results of the secondary data user survey, especially the statistical part, are therefore relevant for German academic institutions.

### 2.3 Limitations

Our methodological approach goes along with common limitations of systematic reviews and qualitative methods. The sample of papers in the systematic review is limited to journal publications in well-known databases and excludes for example monographs, grey literature, and papers from public repositories such as preprints. Our sample does in this regard draw a picture of specific scope, leaving out for instance texts from open data initiatives or blog posts. Systematic reviews are furthermore prone to publication-bias [19] the tendency to publish positive results rather than negative results. We tried to counteract a biased analysis by triangulating the derived category system with empirical data from a survey among secondary data users [20]. For the analysis, we leaned onto quality criteria of qualitative research. Regarding the validity of the identified categories, an additional quantitative survey is recommended.

### 2.4 Ethics Statement

The SOEP User Survey was approved by the data protection officer of the German Institute for Economic Research (DIW Berlin) and the head of the research data center DIW-SOEP. The qualitative answers have been made available without personal data to guarantee the interviewees’ anonymity.

## Results

**Fig 4:**
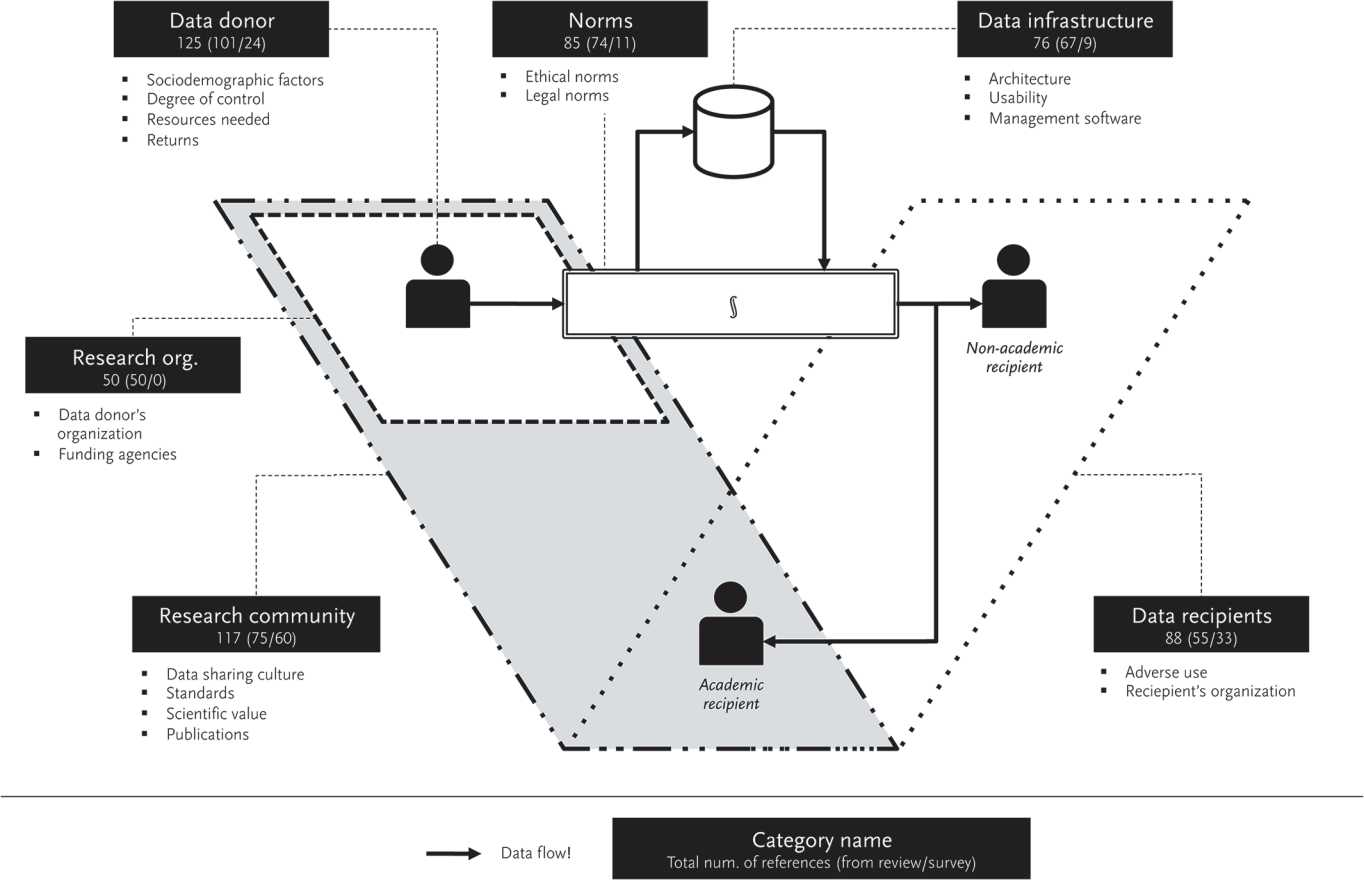
Conceptional framework for academic data sharing.

As a result of the systematic review and the survey we arrived at a framework that depicts academic data sharing in six descriptive categories. Fig. 4 provides an overview of these six (*data donor, research organization, research community, norms, data infrastructure*, and *data recipients*) and highlights how often we found references for them in a) the literature review and b) in the survey (a/b). In total we found 541 references, 404 in the review and 137 in our survey. Furthermore, the figure shows the subcategories of each category.

- *Data donor*, comprising factors regarding the individual researcher who is sharing data (e.g., invested resources, returns received for sharing)
- *Research organization*, comprising factors concerning the crucial organizational entities for the donating researcher, being the own organization and funding agencies (e.g., funding policies)
- *Research community*, comprising factors regarding the disciplinary data-sharing practices (e.g., formatting standards, sharing culture)
- *Norms*, comprising factors concerning the legal and ethical codes for data sharing (e.g., copyright, confidentiality)
- *Data recipients*, comprising factors regarding the third party reuse of shared research data (e.g., adverse use)
- *Data infrastructure*, comprising factors concerning the technical infrastructure for data sharing (e.g., data management system, technical support)

In the following, we explain the hindering and enabling factors for each category. Each category is summarized by a table that lists the identified sub-categories and data sharing factors. The tables further provide text references and direct quotes for selected factors. We translated most of the direct quotes from the survey from German to English, as 76% the respondents are German.

### 3.1 Data Donor

The category *data donor* concerns the individual researcher who collects data. The sub-categories are *sociodemographic factors, degree of control, resources needed*, and *returns* (see Table 4).

**Table 4:** Overview category data donor.

**Sub-category**	**Factors**	**References**	**Exemplary Quotes**
Sociodemographic factors	Nationality, Age, Seniority and career prospects, Character traits, Research practice	Acord and Harley, 2012; Enke et al., 2012; Milia et al., 2012; Piwowar, 2011; Tenopir et al., 2011	*Age*: “There are some differences in responses based on age of respondent. Younger people are less likely to make their data available to others (either through their organization’s website, PI’s website, national site, or other sites.). People over 50 showed more interest in sharing data (…)” (Tenopir et al. 2011, p. 14)
Degree of control	Knowledge about data requester, Having a say in the data use, Priority rights for publications	Acord and Harley, 2012; Belmonte et al., 2007; Bezuidenhout, 2013; Constable et al., 2010; Enke et al., 2012; Fisher and Fortman, 2010; Huang et al., 2013; Jarnevich et al., 2007; Pearce and Smith, 2011; Pitt and Tang, 2013; Stanley and Stanley, 1988; Tenopir et al., 2011; Whitlock et al., 2010; Wallis et al., 2013	*Having a say in the data use*: “I have doubts about others being able to use my work without control from my side” (Survey)
Resources needed	Time and effort, Skills and knowledge, Financial resources	Acord and Harley, 2012; Axelsson and Schroeder, 2009; Breeze et al., 2012; Campbell et al., 2002; Cooper, 2007; Costello, 2009; De Wolf et al., 2006; De Wolf et al., 2005; Enke et al., 2012; Gardner et al., 2003; Constable and Guralnick, 2010; Haendel et al., 2012; Huang et al., 2013; Kowlcyk and Shankar, 2013; Nelson, 2009; Noor et al., 2006; Perrino et al., 2013; Piwowar et al., 2008; Reidpath and Allotey, 2001; Rushby, 2013; Savage and Vickers, 2009; Sayogo and Pardo, 2013; Sieber, 1988; Stanley and Stanley, 1988; Teeters et al., 2008; Van Horn and Gazzaniga, 2013; Wallis et al., 2013; Wicherts and Bakker, 2006	*Time and effort*: “The effort to collect data is immense. To collect data yourself becomes almost out of fashion.” (Survey)
Returns	Formal recognition, Professional exchange, Quality improvement	Acord and Harley, 2012; Costello, 2009, Dalgleish et al., 2012; Elman et al., 2010; Enke et al., 2012; Gardner et al., 2003; Mennes et al., 2013; Nelson, 2009; Ostell, 2009; Parr, 2007; Perrino et al., 2013; Piwowar et al., 2007; Reidpath and Allotey, 2001; Rohlfing and Poline, 2012; Stanley and Stanley, 1988; Tucker, 2009; 1988; Wallis et al., 2013; Wicherts and Bakker, 2012; Whitlock, 2011	*Formal recognition*: “… the science reward system has not kept pace with the new opportunities provided by the internet, and does not sufficiently recognize online data publication.” (Costello, 2009, p. 426)

### 3.1.1 Sociodemographic factors

Frequently mentioned in the literature were the factors *age, nationality*, and *seniority in the academic system*. Enke et al. [21], for instance, observe that German and Canadian scientists were more reluctant to share research data publicly than their US colleagues (which raises the question how national research policies influence data sharing). Tenopir et al. [5] found that there is an influence of the researcher’s *age* on the willingness to share data. Accordingly, younger people are less likely to make their data available to others. People over 50, on the other hand, were more likely to share research data. This result resonates with an assumed influence of *seniority in the academic system* and competitiveness on data sharing behavior [22]. Data sets and other subsidiary products are awarded far less credit in tenure and promotion decisions than text publications [23]. Hence does competition, especially among non-tenured researchers, go hand in hand with a reluctance to share data. The perceived *right to publish first* (see *degree of control*) with the data further indicates that publications and not (yet) data is the currency in the academic system. Tenopir et al. (2011) [5] point to an influence of the level of *research activity* on the willingness to share data. Individuals who work solely in research, in contrast to researchers who have time-consuming teaching obligations, are more likely to make their data available to other researchers. Acord and Harley [23] further regard *character traits* as an influencing factor. This conjecture is not vindicated in our questionnaire. In contrast to our initial expectations, character traits (Big Five) are not able to explain much of the variation in *Q1*. In a logistic regression model on the willingness to share data and scripts in general (answer categories 1–3) and controlling for age and gender, only the *openness* dimension shows a significant influence *(positive influence with p < 0.005)*. All other dimensions (*conscientiousness, extraversion, agreeableness, neuroticism*) do not have a considerable influence on the willingness to share.

### 3.1.2 Degree of control

A core influential factor on the individual data sharing behavior can be subsumed under the category *degree of control*. It denotes the researcher’s need to *have a say* or at least *knowledge* regarding the access and use of the deposited data.

The relevance of this factor is emphasized by the results to question Q1 in our survey (see Table 5). Only a small number of researchers (18%) categorically refuses to share scripts or research data. For those who are willing to share (82%), control seems to be an important issue (summarized by the first three questions). 56% are either demanding a context with access control or would only be willing to share on request. However, it has to be said that our sample comprises mostly German-speaking researchers that are familiar with secondary data and is therefore not representative for the academia in general.

**Table 5:** Question on sharing analysis scripts and data sets.

**Sharing analysis scripts and data sets**	**Freq**	**Perc (valid)**
1. Willing to share publicly	120	25.9%
2. Willing to share under access control	98	21.1%
3. Willing to share only on request	163	35.1%
4. Not willing	83	17.9%
***Sum***	***464***	***100%***

*Source*: *SOEP USer Survey 2013, own calculations*

Eschenfelder and Johnson [24] suggest more control for researchers over deposited data (see also [25–32]). According to some scholars, a *priority right* for publications, for example an embargo on data (e.g.,[33]), would enable academic data sharing. Other authors point to a researcher’s concern regarding the technical ability of the data requester to understand [29,34] and to interpret [35] a dataset (see also data recipients→adverse use). The need for control is also present in our survey among secondary data users. To the question why one would not share research data, one respondent replied: “I have doubts about others being able to use my work without control from my side” (Survey). Another respondent replied: “I want to know who uses my data.” (Survey). The results in this category indicate a perceived ownership over the data on the part of the researcher, which is legally often not the case.

### 3.1.3 Resources needed

Here we subsume factors relating to the researcher’s investments in terms of *time* and *costs* as well as their *knowledge* regarding data sharing. “Too much effort!” was a blunt answer we found in our survey as a response to the question why researchers do not share data. In the literature we found the argument *time and effort* 19 times and seven times in the survey. One respondent stated: “The effort to collect data yourself is immense and seems "not to be in fashion" anymore. I don't want to support this convenience” (from the survey). Another respondent said “(the) amount of extra work to respond to members of the research community who either want explanation, support, or who just want to vent." would prevent him or her from sharing data. Besides the actual sharing effort [21,23,29,33,36–41], scholars utter concerns regarding the effort required to help others to make sense of the data [42]. The *knowledge* factor becomes apparent in Sieber’s study [43] in which most researchers stated that data sharing is advantageous for science, but that they had not thought about it until they were asked for their opinion. Missing knowledge further relates to poor curation and storing skills [33,44] and missing knowledge regarding adequate repositories [21,42]. In general, missing knowledge regarding the existence of databases and know-how to use them is described as a hindering factor for data sharing. Several scholars, for instance Piwowar et al. [45] and Teeters et al.[46], hence suggest to integrate data sharing in the curriculum. Others mention the *financial effort* to share data and suggest forms of financial compensation for researchers or their organizations [43,47].

### 3.1.4 Returns

Within the examined texts we found 26 references that highlight the issue of missing returns in exchange for sharing data, 12 more came from the survey. The basic attitude of the references describes a lack of *recognition* for data donors [29,31,48–51]. Both sources – review and survey – argue that donors do not receive enough formal *recognition* to justify the individual efforts and that a safeguard against uncredited use is necessary [36,52–54]. The form of attribution a donor of research data should receive remains unclear and ranges from a mentioning in the acknowledgments to citations and co-authorships [21]. One respondent stated: “"It is your own effort that is taken by others without citation or reward" (from the survey). Several authors explain that impact metrics need to be adapted to foster data sharing [34,55]. Yet, there is also literature that reports positive individual returns from shared research data. Kim and Stanton [56] for instance explain that shared data can highlight the quality of a finding and thus indicate sophistication. Piwowar et al. [45] report an increase in citation scores for papers, which feature supplementary data. Further, *quality improvements* in the form of *professional* are mentioned: “Seeing how others have solved a problem may be useful.”, “I can profit and learn from other people’s work.”, “to receive feedback from other researchers and to make my analysis repeatable.” (all quotes are from our survey). Enke et al. [21] also mention an increased visibility within the research community as a possible positive return.

### 3.2 Research Organization

The category *research organization* comprises the most relevant organizational entities for the donating researcher. These are the data donor’s own organization as well as funding agencies (see Table 6).

**Table 6:** Overview category research organization.

**Sub-category**	**Factors**	**References**	**Exemplary Quotes**
Data donor’s organization	Data sharing policy and organizational culture, Data management	Belmonte et al., 2013; Breeze et al., 2012; Cragen et al., 2010; Enke et al., 2012; Huang et al., 2013; Masum et al., 2013; Pearce and Smith, 2011; Perrino et al., 2013; Savage and Vickers, 2009; Sieber, 1988	*Data sharing policy and organizational culture*: “Only one-third of the respondents reported that sharing data was encouraged by their employers or funding agencies.” (Huang et al., 2013, p. 404)
Funding agencies	Funding policy (grant requirements), Financial compensation	Axelsson and Schroeder, 2009; Borgman, 2012; Eisenberg, 2006; Enke et al., 2012; Cohn, 2012; Fernandez et al., 2012; Huang et al., 2013; Mennes et al., 2013; Nelson, 2009; NIH, 2002; NIH, 2003; Perrino et al., 2013; Pitt and Tang, 2013, Piwowar et al., 2008; Sieber, 1988; Stanley and Stanley, 1988; Teeters et al., 2008; Wallis et al., 2013; Wicherts and Bakker, 2012	*Funding policy (grant requirements)*: “… until data sharing becomes a requirement for every grant […] people aren’t going to do it in as widespread of a way as we would like.” (Nelson, 2009, p. 161)

### 3.2.1 Data donor’s organization

An individual researcher is generally placed in an organizational context, for example a university, a research institute or a research and development department of a company. The respective organizational affiliation can impinge on his or her data sharing behaviour especially through internal policies, the organizational culture as well as the available data infrastructure. Huang et al. [35] for instance, in an international survey on biodiversity data sharing found out that “only one-third of the respondents reported that sharing data was encouraged by their employers or funding agencies”. The respondents whose organizations or affiliations encourage data sharing were more willing to share. Huang et al. [35] view the *organizational policy* as a core adjusting screw. They suggest detailed data management and archiving instructions as well as recognition for data sharing (i.e., career options). Belmonte et al. [57] and Enke et al. [21] further emphasize the importance of intra-organizational *data management*, for instance consistent data annotation standards in laboratories (see also *3.6. Data Infrastructure*). Cragin et al. [58] see data sharing rather as a community effort in which the single organizational entity plays a minor role: “As a research group gets larger and more formally connected to other research groups, it begins to function more like big science, which requires production structures that support project coordination, resource sharing and increasingly standardized information flow”.

### 3.2.2 Funding agencies

Besides journal policies, the *policies* of funding agencies are named as a key adjusting screw for academic data sharing throughout the literature (e.g.,[21,42]). Huang et al. [35] argue that making data available is no obligation with many funding agencies and that they do not provide sufficient *financial compensation* for the efforts needed in order to share data. Perrino et al. [59] argue that funding policies show varying degrees of enforcement when it comes to data sharing and that binding policies are necessary to convince researchers to share. The National Science Foundation of the US, for instance, has long required data sharing in its grant contracts (see [60]), “but has not enforced the requirements consistently” [61].

### 3.3 Research Community

The category research community subsumes the sub-categories data sharing culture, standards, scientific value, and publications (see Table 7).

**Table 7:** Overview category research community.

**Sub-category**	**Factors**	**References**	**Exemplary Quotes**
Data sharing culture	Disciplinary practice	Costello, 2009; Enke et al., 2012; Haendel et al., 2012; Huang et al., 2013; Milia et al., 2012; Nelson, 2009; Tenopir et al., 2011	*Disciplinary practice*: “The main obstacle to making more primary scientific data available is not policy or money but misunderstandings and inertia within parts of the scientific community.” (Costello, 2009, p. 419)
Standards	Metadata, Formatting standards, Interoperability	Axelsson and Schroeder, 2009; Costello, 2009; Delson et al., 2007; Edwards et al., 2011; Enke et al., 2012; Freymann et al., 2012; Gardner et al., 2003; Haendel et al., 2012; Jiang et al., 2013; Jones et al., 2012; Linkert et al., 2010; Milia et al., 2012; Nelson, 2009; Parr, 2007; Sansone and Rocca-Serra, 2012; Sayogo and Pardo, 2013; Teeters et al., 2008; Tenopir et al., 2011; Wallis et al., 2013; Whitlock, 2011	*Metadata*: “In our experience, storage of binary data […] is based on common formats […] or other formats that most software tools can read […]. The much more challenging problem is the metadata. Because standards are not yet agreed upon.” (Linkert et al., 2010, p. 779)
Scientific value	Scientific progress, Exchange, Review, Synergies	Alsheikh-Ali et al., 2011; Bezuidenhout, 2013; Butler, 2007, Chandramohan et al., 2008; Chokshi et al., 2006; Costello, 2009; De Wolf et al., 2005; Huang et al., 2013; Ludman et al., 2010; Molloy, 2011; Nelson, 2009; Perrino et al., 2013; Pitt and Tang, 2013; Piwowar et al., 2008; Stanley and Stanley, 1988; Teeters et al., 2008; Tenopir et al., 2011; Wallis et al., 2013; Whitlock et al., 2010	*Exchange*: “The main motivation for researchers to share data is the availability of comparable data sets for comprehensive analyses (72%), while networking with other researchers (71%) was almost equally important.” (Enke et al., 2012, p. 28)
Publications	Journal policy	Alsheikh-Ali et al., 2011; Anagnostou et al., 2013; Chandramohan et al., 2008; Costello, 2009; Enke et al., 2012; Huang et al., 2012; Huang et al., 2013; Milia et al., 2012; Noor et al., 2006; Parr, 2007; Pearce and Smith, 2011; Piwowar, 2010; Piwowar, 2011; Piwowar and Chapman, 2010; Tenopir et al., 2011; Wicherts et al., 2006; Whitlock 2011	*Data sharing policy*: “It is also important to note that scientific journals may benefit from adopting stringent sharing data rules since papers whose datasets are available without restrictions are more likely to be cited than withheld ones.” (Milia et al., 2012, p. 3)

### 3.3.1 Data sharing culture

The literature reports a substantial variation in academic data sharing across *disciplinary practices* [22,62]. Even fields, which are closely related like medical genetics and evolutionary genetics show substantially different sharing rates [22]. Medical research and social sciences are reported to have an overall low data sharing culture [5], which possibly relates to the fact that these disciplines work with individual-related data. Costello [34] goes so far as to describe the data sharing culture as the main obstacle to academic data sharing (see Table 7 for quote). Some researchers see the community culture rather as a motivation. To the question what would motivate a researcher to share data, for instance, one respondent replied: “to extend the community of data users in my research community“, or „Everyone benefits from sharing data if you don't have to reinvent the wheel“(from the survey).

### 3.3.2 Standards

When it comes to the interoperability of data sets, many scholars see the absence of *metadata* standards and *formatting standards* as an impediment for sharing and reusing data; lacking standards hinder *interoperability* (e.g., [5,21,34,46,55,62–68]. There were no references in the survey for the absence of formatting standards.

### 3.3.3. Scientific value

In this subcategory we subsume all findings that bring value to the scientific community. It is a very frequent argument that data sharing can enhance *scientific progress*. A contribution hereto is often considered an intrinsic motivation for participation. This is supported by our survey: We found sixty references for this subcategory, examples are “Making research better”, “Feedback and exchange”, “Consistency in measures across studies to test robustness of effect”, “Reproducibility of one’s own research”. Huang et al. [35] report that 90% of their “respondents indicated the desire to contribute to scientific progress” [69]. Tenopir et al. report that “(67%) of the respondents agreed that lack of access to data generated by other researchers or institutions is a major impediment to progress in science” [5]. Other scholars argue that data sharing accelerates scientific progress because it helps find *synergies* and avoid repeating work [42,70–76]. It is also argued that shared data increases quality assurance and makes the *review* process better and that it increases the networking and the *exchange* with other researchers [21]. Wicherts and Bakker [77] argue that researchers who share data commit less errors and that data sharing encourages more research (see also [78]).

### 3.3.4 Publications

In most research communities publications are the primary currency. Promotions, grants, and recruitments are often based on publications records. The demands and offers of publication outlets therefore have an impact on the individual researcher’s data sharing disposition [79]. Enke et al. [21]describe *journal policies* to be the major motivator for data sharing, even before funding agencies. A study conducted by Huang et al. [69] shows that 74% of researchers would accept leading journals’ data sharing policies. However, other research indicates that today’s journal policies for data sharing are all but binding [5,27,80,81]. Several scholars argue that more stringent data sharing policies are needed to make researchers share [27,55,69,82]. At the same time they argue that publications that include or link to the used dataset receive more citations. And therefore both journals and researchers should be incentivised to follow data sharing policies[55].

### 3.4 Norms

In the category *norms* we subsume all ethical and legal codes that impact a researcher’s data sharing behaviour (see Table 8).

**Table 8:** Overview category norms.

**Sub-category**	**Factors**	**References**	**Exemplary Quotes**
Ethical norms	Confidentiality, Informed consent, Potential harm	Axelsson and Schroeder, 2009; Brakewood and Poldrack, 2013; Cooper, 2007; De Wolf et al., 2006; Fernandez et al., 2012; Freymann et al., 2012; Haddow et al., 2011; Jarnevich et al., 2007; Levenson, 2010; Ludman et al., 2010; Mennes et al., 2013; Pearce and Smith, 2011; Perrino et al., 2013; Resnik, 2010; Rodgers and Nolte, 2006; Sieber, 1988; Tenopir et al., 2011	*Informed consent*: “Autonomous decision-making means that a subject [patient] needs to have the ability to think about his or her choice to participate or not and the ability to actually act on that decision.” (Brakewood and Poldrack, 2013, p. 673)
Legal norms	Ownership and right of use, Privacy, Contractual consent, Copyright	Axelsson and Schroeder, 2009; Brakewood and Poldrack, 2013; Breeze et al., 2012; Cahill and Passamo, 2007; Cooper, 2007; Costello, 2009; Chandramohan et al., 2008; Chokshi et al., 2006; Dalgleish et al., 2012; De Wolf et al., 2005; De Wolf et al., 2006; Delson et al., 2007; Eisenberg, 2006; Enke et al., 2012; Freymann et al., 2012; Haddow et al., 2011; Kowalczyk and Shankar, 2011; Levenson, 2010; Mennes et al., 2013; Nelson, 2009; Perrino et al., 2013; Pitt and Tang, 2013; Rai and Eisenberg, 2006; Reidpath and Allotey, 2001; Resnik, 2010; Rohlfing and Poline, 2012; Teeters et al., 2008; Tenopir et al., 2011	*Ownership and right of use*: “In fact, unresolved legal issues can deter or restrain the development of collaboration, even if scientists are prepared to proceed.” (Sayogo and Pardo, 2013, p. 21)

### 3.4.1 Ethical norms

As ethical norms we regard moral principles of conduct from the data collector’s perspective. Brakewood and Poldrack [83] regard the respect for persons as a core principle in data sharing and emphasize the importance of informed consent and *confidentiality*, which is particularly relevant in the context of individual-related data. The authors demand that a patient “needs to have the ability to think about his or her choice to participate or not and the ability to actually act on that decision”. De Wolf et al. [84], Harding et al. [85], Mennes et al. [86], Sheather [87] and Kowalczyk and Shankar [88] take the same line regarding the necessity of *informed consent* between researcher and study subject. Axelsson and Schroeder [63] describe the maxim to act upon public trust as an important precondition for database research. Regarding data sensitivity, Cooper [89] emphasizes the need to consider if the data being shared could harm people. Similarly, Enke et al. [21] point to the possibility that some data could be used to *harm* environmentally sensitive areas. Often ethical considerations in the context of data sharing concern adverse use of data, we specify that under adverse use in the category data recipients.

### 3.4.2 Legal norms

Legal uncertainty can deter data sharing, especially in disciplines that work with sensitive data, example are corporate or personal data [90]. Under legal norms we subsume *ownership and rights of use, privacy, contractual consent* and *copyright*. These are the most common legal issues regarding data sharing. The sharing of data is restricted by the national privacy acts. In this regard, Freyman et al. [91] and Pitt and Tang [30] emphasize the necessity for de-identification as a pre-condition for sharing individual-related data. Pearce and Smith [27] on the other hand state that getting rid of identifiers is often not enough and pleads for restricted access. Many authors point to the necessity of *contractual consent* between data collector and study participant regarding the terms of use of personal data [25,47,84,86,89,92–96]. While *privacy* issues apply to individual-related data, issues of *ownership and rights of use* concern all kinds of data. Enke et al. [21] states that the legal framework concerning the ownership of research data before and after deposition in a database is complex and involves many uncertainties that deter data sharing (see also [34,97]). Eisenberg [98] even regards the absence of adequate intellectual property rights, especially in the case of patent-relevant research, as a barrier for data sharing and therefore innovation (see also [22,99]). Chandramohan et al. [100] emphasize that data collection financed by tax money is or should be a public good.

### 3.5 Data Recipient

In the category data recipients, we subsumed influencing factors regarding the use of data by the data recipient and the recipient’s organizational context (see Table 9).

**Table 9:** Overview category data recipients.

**Sub-category**	**Factors**	**References**	**Exemplary Quotes**
Adverse Use	Falsification, Commercial misuse, Competitive misuse, Flawed interpretation, Unclear intent	Acord and Harley, 2012; Anderson and Schonfeld, 2009; Cooper, 2007; Costello, 2009; De Wolf et al., 2006; Enke et al., 2012; Fisher and Fortman, 2010; Gardner et al., 2003; Harding et al., 2011; Hayman et al., 2012; Huang et al., 2012; Huang et al., 2013; Molloy, 2011; Nelson, 2009; Overbey, 1999; Parr and Cummings, 2005; Pearce and Smith, 2011; Perrino et al., 2013; Reidpath and Allotey, 2001; Sieber, 1988; Stanley and Stanley, 1988; Tenopir et al., 2011; Wallis et al., 2013; Zimmerman, 2008	*Falsification*: “I am afraid that I made a mistake somewhere that I didn’t find myself and someone else finds.” (Survey)
			*Competitive Use*: “Furthermore I have concerns that I used my data exhaustively before I publish it.” (Survey)
Recipient’s organization	Data security conditions, Commercial or public organization	Fernandez et al., 2012; Tenopir et al., 2011	*Data security conditions*: “Do the lab facilities of the receiving researcher allow for the proper containment and protection of the data? Do the […] security policies of the receiving lab/organization adequately reduce the risk that an internal or external party accesses and releases the data in an unauthorized fashion?” (Fernandez et al., 2012, p. 138)

### 3.5.1 Adverse use

A multitude of hindering factors for data sharing in academia can be assigned to a presumed *adverse use* on the part of the data recipient. In detail, these are *falsification, commercial misuse, competitive misuse, flawed interpretation*, and *unclear intent*. For all of these factors, references can be found in both, the literature and the survey. Regarding the fear of *falsification*, one respondent states: “I am afraid that I made a mistake somewhere that I didn’t find myself and someone else finds.” In the same line, Costello [34] argues that “[a]uthors may fear that their selective use of data, or possible errors in analysis, may be revealed by data publication”. Many authors describe the fear of falsification as a reason to withhold data [23,27,29,34,43,101]. Few authors see a potential “*commercialization* of research findings” [93] as a reason not to share data (see also: [34,36,92]). The most frequently mentioned withholding reason regarding the third party use of data is *competitive misuse*; the fear that someone else publishes with *my* data before *I* can (16 survey references, 13 text references). This indicates that at least from the primary researcher’s point of view, withholding data is a common competitive strategy in a publication-driven system. To the question, what concern would prevent one from sharing data, one researcher stated: "I don't want to give competing institutions such far-reaching support." Costello [34] encapsulates this issue: “If I release data, then I may be scooped by somebody else producing papers from them.” Many other authors in our sample examine the issue of *competitive misuse* [5,22,23,46,48,102–104]. Another issue regarding the recipient’s use of data concerns a possible *flawed interpretation* (e.g., [21,59,89,105,106]). Perrino et al. [59], regarding a dataset from psychological studies, state: “The correct interpretation of data has been another concern of investigators. This included the possibility that the [data recipient] might not fully understand assessment measures, interventions, and populations being studied and might misinterpret the effect of the intervention”. The issue of flawed interpretation is closely related to the factor data documentation (see *3.6 data infrastructure*). Associated with the need for control (as outlined in *data donor*), authors and respondents alike mention a declaration of intent as an enabling factor, as one of the respondents states “missing knowledge regarding the purpose and the recipient” is a reason not to share (see also). Respondents in the survey stated that sharing data would lead to more transparency: “(I would share data) to benefit from others' scripts and for transparency in research“(from the survey).

### 3.5.2 Recipient’s organization

According to Fernandez et al. [107] the recipient’s organization, its type (*commercial or public*) and *data security conditions*, have some impact on academic data sharing. Fernandez et al. [107] summarize the potential uncertainties: “Do the lab facilities of the receiving researcher allow for the proper containment and protection of the data? Do the physical, logical and personnel security policies of the receiving lab/organization adequately reduce the risk that [someone] release[s] the data in an unauthorized fashion?” (see also [5]).

### 3.6 Data Infrastructure

In the category data infrastructure we subsume all factors concerning the technical infrastructure to store and retrieve data. It is comprised of the sub-categories *architecture, usability*, and *management software (see Table 10)*.

**Table 10:** Overview category data infrastructure.

**Sub-category**	**Factors**	**References**	**Exemplary Quotes**
Architecture	Access, Performance, Storage, Data quality, Data security	Axelsson and Schroeder, 2009; Constable et al., 2010; Cooper, 2007; De Wolf et al., 2006; Enke et al., 2012; Haddow et al., 2011; Huang et al., 2012; Jarnevich et al., 2007; Kowalczyk and Shankar, 2011; Linkert et al., 2010; Resnik, 2010; Rodgers and Nolte, 2006; Rushby, 2013; Sayogo and Pardo, 2013; Teeters et al., 2008	*Access*: “It is not permitted, for example, for a faculty member to obtain the data for his or her own research project and then “lend” it to a graduate student to do related dissertation research, even if the graduate student is a research staff signatory, unless this use is specifically stated in the research plan.” (Rodgers and Nolte, 2006, p. 90)
Usability	Tools and applications, Technical support	Axelsson and Schroeder, 2009; Haendel et al., 2012; Mennes et al., 2013; Nicholson and Bennett, 2011; Ostell, 2009; Teeters et al., 2008	*Tools and applications*: “At the same time, the platform should make it easy for researchers to share data, ideally through a simple one-click upload, with automatic data verification thereafter.” (Mennes et al. 2013, p. 688)
Management software	Data documentation, Metadata standards	Acord and Hartley, 2012; Axelsson and Schroeder, 2009; Breese et al., 2012; Constable et al., 2010; Delson et al., 2007; Edwards et al., 2011; Enke et al., 2012; Gardner et al., 2003; Jiang et al., 2013; Jones et al., 2012; Karami et al., 2013; Linkert et al., 2010; Myneni and Patel, 2010; Nelson, 2009; Parr, 2007; Sansone and Rocca-Serra, 2012; Teeters et al., 2008; Tenopir et al., 2011	*Data documentation*: “The authors came to the conclusion that researchers often fail to develop clear, well-annotated datasets to accompany their research (i.e., metadata), and may lose access and understanding of the original dataset over time.” (Tenopir et al. 2011, p. 2)

### 3.6.1 Architecture

A common rationale within the surveyed literature is that restricted access, for example through a registration system, would contribute to *data security* and counter the perceived loss of control ([47,87,89,94,108]). Some authors even emphasize the necessity to edit the data after it has been *stored* [21]. There is however disunity if the infrastructure should be centralized (e.g., [65]) or decentralized (e.g.,[109]). Another issue is that *data quality* is maintained after it has been archived [83]. In this respect, Teeters et al. [46] suggest that data infrastructure should provide technical support and means of indexing.

### 3.6.2 Usability

The topic of usability comes up multiple times in the literature. The authors argue that service providers need to make an effort to simplify the sharing process and the involved *tools* [46,86]. Authors also argue that guidelines are needed besides a *technical support* that make it easy for researchers to share [110,111].

### 3.6.3 Management system

We found 24 references for the *management system* of the *data infrastructure*, these were concerned with *data documentation* and *metadata standards* [5,63,65,112]. The *documentation* of data remains a troubling issue and many disciplines complain about missing standards [23,113,114]. At the same time other authors explain that detailed *metadata* is needed to prevent misinterpretation [36].

## Discussion and Conclusion

The accessibility of research data holds great potential for scientific progress. It allows the verification of study results and the reuse of data in new contexts ([1,2]). Despite its potential and prominent support, sharing data is not yet common practice in academia. With the present paper we explain that data sharing in academia is a multidimensional effort that includes a diverse set of stakeholders, entities and individual interests. To our knowledge, there is no overarching framework, which puts the involved parties and interests in relation to one another. In our view, the conceptual framework with its empirically revised categories has theoretical and practical use. In the remaining discussion we will elaborate possible implications for theory, and research practice. We will further address the need for future research.

### 4.1 Theoretical Implications: Data is Not a Knowledge Commons

Concepts for the production of immaterial goods in a networked society frequently involve the dissolution of formal entities, modularization of tasks, intrinsic motivation of participants, and the absence of ownership. Benkler’s [6] *commons-based peer production*, to a certain degree *wisdom of the crowds* [115] and *collective intelligence* [116] are examples for organizational theories that embraces novel forms of networked collaboration. Frequently mentioned empirical cases for these forms of collaboration are the open source software community [117] or the online encyclopedia Wikipedia [118,119]. In both cases, the product of the collaboration can be considered a commons. The production process is inherently inclusive.

In many respects, Franzoni and Sauermann’s [7] theory for *crowd science* resembles the concepts commons-based peer production and crowd intelligence. The authors dissociate crowd science from traditional science, which they describe as largely closed. In traditional science, researchers retain exclusive use of key intermediate inputs, such as data. Crowd science on the other hand is characterized by its inherent openness with respect to participation and the disclosure of intermediate inputs such as data. Following that line of thought, data could be considered a knowledge commons, too. A good that can be accessed by everyone and whose consumption is non-rivalry [120]. Crowd-science is in that regard a commons-driven principle for scholarly knowledge. In many respects, academia indeed fulfils the requirements for crowd science, be it the immateriality of knowledge products, the modularity of research, and public interest.

In the case of data sharing in academia, however, the theoretical depiction of a crowd science [7] or an open science [121], and with both the accessibility of data, does not meet the empirical reality. The core difference to the model of a commons-based peer production like we see it in open source software or crowd science lies in the motivation for participation. Programmers do not have to release their code under an open source licence. And many do not. The same is true for Wikipedia, where a rather small but active community edits pages. Both systems run on voluntariness, self-organization, and intrinsic motivation. Academia however, contradicts to different degrees these characteristics of a knowledge exchange system.

In an ideal situation every researcher would publish data alongside a research paper to make sure the results are reproducible and the data is reusable in a new context. Yet today, most researchers remain reserved to share their research data. This indicates that their efforts and perceived risks outweigh the potential individual benefits they expect from data sharing. Research data is in large parts not a knowledge commons. Instead, our results points to a *perceived ownership of data* (reflected in the right to publish first) and a *need for control* (reflected in the fear of data misuse). Both impede a commons-based exchange of research data. When it comes to data, academia remains neither accessible nor participatory. As data publications lack sufficient *formal recognition* (e.g., citations, co-authorship) in comparison to text publications, researchers find furthermore too few incentives to share data. While altruism and with it the idea to contribute to a common good, is a sufficient driver for some researchers, the majority currently remains incentivised not to share. If data sharing leads to better science and simultaneously, researchers are hesitant to share, the question arises how research policies can foster data sharing among academic researchers.

### 4.2 Policy Implications: Towards more Data Sharing

Worldwide, research policy makers support the accessibility of research data. This can be seen in the US with efforts by the National Institutes of Health [122,123] and also in Europe, with the EU’s Horizon 2020 programme [3]. In order to develop consequential policies for data sharing, policy makers need to understand and address the involved parties and their perspectives. The framework that we present in this paper helps to gain a better understanding of the prevailing issues and provides insights into the underlying dynamics of academic data sharing. Considering that research data is far from being a commons, we believe that research policies should work towards an efficient exchange system in which as much data is shared as possible. Strategic policy measures could therefore go into two directions: First, they could provide incentives for sharing data and second impede researchers not to share. Possible incentives could include adequate *formal recognition* in the form of data citation and career prospects. In academia, a largely non-monetary knowledge exchange system, research policy should be geared towards making intermediate products count more. Furthermore could forms of *financial reimbursement*, for example through additional person hours in funding, help to increase the individual effort to make data available. As long as academia remains a publication-centred business, journal policies further need to adopt mandatory data sharing policies [2,124] and provide easy-to use data management systems. Impediments regarding sharing supplementary data could include clear and elaborate reasons to opt out. In order to remove risk aversion and ambiguity, an understandable and clear legal basis regarding the rights of use is needed to inform researchers on what they can and cannot do with data they collected. This is especially important in medicine and in the social sciences where much data comes from individuals. Clear guidelines that explain how consent can be obtained and how data can be anonymized are needed. Educational efforts within data-driven research fields on proper data curation, sharing culture, data documentation, and security could be fruitful in the intermediate-term. Ideally these become part of the curriculum for university students. Infrastructure investments are needed to develop efficient and easy-to-use data repositories and data management software, for instance as part of the Horizon 2020 research infrastructure endeavors.

### 4.3 Future Research

We believe that more research needs to address the discipline-specific barriers and enablers for data sharing in academia in order to make informed policy decisions. Regarding the framework that we introduce in this paper, the identified factors need further empirical revision. In particular, we regard the intersection between academia and industry worth investigating. For instance: A study among German life scientists showed that those who receive industry funding are more likely to deny others’ requests for access to research materials [125]. In the same line, Haeussler [126] in a comparative study on information sharing among scientists, finds that the likelihood of sharing information decreases with the competitive value of the requested information. It increases when the inquirer is an academic researcher. Following this, future research could address data sharing between industry and academia. Open enterprise data, for example, appears to be a relevant topic for legal scholars as well as innovation research.
